# Local anesthesia in piglets undergoing castration—A comparative study to investigate the analgesic effects of four local anesthetics on the basis of acute physiological responses and limb movements

**DOI:** 10.1371/journal.pone.0236742

**Published:** 2020-07-30

**Authors:** Anna M. Saller, Julia Werner, Judith Reiser, Steffanie Senf, Pauline Deffner, Nora Abendschön, Christine Weiß, Johannes Fischer, Andrea Schörwerth, Regina Miller, Yury Zablotski, Shana Bergmann, Michael H. Erhard, Mathias Ritzmann, Susanne Zöls, Christine Baumgartner

**Affiliations:** 1 Center of Preclinical Research, Technical University of Munich, Bavaria, Germany; 2 Clinic for Swine, Center for Clinical Veterinary Medicine, LMU Munich, Bavaria, Germany; 3 Chair of Animal Welfare, Ethology, Animal Hygiene and Husbandry, LMU Munich, Bavaria, Germany; Max Delbruck Centrum fur Molekulare Medizin Berlin Buch, GERMANY

## Abstract

Surgical castration of male piglets without analgesia is a painful procedure. This prospective, randomized and double-blinded study aimed to evaluate the analgesic effects of four different local anesthetics for piglet castration during the first week of life. In total, 54 piglets aged 3 to 7 days were distributed into 6 treatment groups: handling (H); castration without pain relief (sodium chloride, NaCl); and castration with a local anesthetic: 4% procaine (P), 2% lidocaine (L), 0.5% bupivacaine (B) or 20 mg/ml mepivacaine (M). By excluding stress and fear as disruptive factors via a minimum anesthesia model, all piglets received individual minimum alveolar concentration (MAC) isoflurane anesthesia. Twenty minutes before castration, all treatment groups except group H received one injection per testis. Then, 0.5 ml of a local anesthetic or NaCl was injected intratesticularly (i.t.), and 0.5 ml was administered subscrotally. Acute physiological responses to noxious stimuli at injection and castration were evaluated by measuring blood pressure (BP), heart rate (HR), cortisol, epinephrine, norepinephrine and chromogranin A (CgA); limb movements were quantified. The results confirm that castration without analgesia is highly painful. Surgical castration without pain relief revealed significant changes in mean arterial blood pressure (MAP) and HR. Local anesthetic administration significantly reduced changes in BP and HR associated with castration. Piglets receiving a preoperative local anesthetic exhibited the fewest limb movements during castration, while the NaCl group exhibited the most. Injection itself was not associated with significant changes in MAP or HR. However, many piglets exhibited limb movements during injection, indicating that the injection itself causes nociceptive pain. No significant differences were found between groups regarding parameters of plasma cortisol, catecholamines and CgA. In conclusion, all four local anesthetics administered are highly effective at reducing signs of nociception during castration under light isoflurane anesthesia. However, injection of a local anesthetic seems to be painful.

## Introduction

In the EU, more than 100 million male piglets are castrated every year to prevent the risk of boar taint in carcasses [1]. Until now in Germany, castration has traditionally been performed without anesthesia in the first week of life, although it has been scientifically shown that surgical castration is a painful procedure [2,3]. Therefore, according to the German Animal Welfare Act, surgical castration of male piglets less than seven days of age without anesthesia will be forbidden in 2021 [4]. Gonadotropin-releasing-hormone (GnRH)-vaccination, boar fattening and surgical castration under general anesthesia are discussed as alternatives to castration without anesthesia. German law also permits the application of local veterinary medicinal products that are able to eliminate pain [4]. However, scientific evidence for the efficacy of local anesthesia for pain relief during castration of piglets is still lacking. For this reason, the discussion about local anesthesia as a legal method in Germany has resurfaced.

As pain sensitivity can differ among different individuals, it is challenging to prove universal pain elimination as requested by German law.

According to the International Association for the Study of Pain (IASP), pain is defined as “an unpleasant sensory and emotional experience associated with actual or potential tissue damage, or described in terms of such damage” [5]. One component of pain is nociception, defined as “the neural process of encoding noxious stimuli” whereby “consequences of encoding may be autonomic (e.g., elevated blood pressure) or behavioral (motor withdrawal reflex or more complex nocifensive behavior)”. The four steps in the development of feeling pain are transduction, transmission, modulation and perception. Nociception mainly refers to the first three components, lacking the conscious awareness and emotional component but including all vegetative, glycemic stress responses and motoric reflex responses, respectively.

In addition to behavioral responses and vocalizations, physiological parameters can be associated with pain [6]. Acute stress, including pain and emotions such as fear and anger, triggers a series of reactions in the sympathetic nervous system [7]. Typical sympathetic reactions that can be measured in pigs are increased blood pressure (BP) and heart rate (HR) and the secretion of catecholamines (epinephrine and norepinephrine) [8,9], and mean arterial blood pressure (MAP) is one of the most sensitive nociceptive parameters in pigs [10]. Another indicator of sympathetic activity is chromogranin A, a neuroendocrine secretory protein that is released together with epinephrine and norepinephrine and is described as a stress marker in pigs [11,12]. Further, serum cortisol levels can be measured to determine castration-induced neuroendocrine stress reactions [13].

Local anesthetics induce anesthesia by blocking sodium channels in the peripheral nerves in the area in which they were administered. Through channel binding, sodium influx into neurons is inhibited, and no action potential is formed [14]. Current studies on castration under local anesthesia show conflicting results. In a study by Kluivers-Poodt et al. [15], piglets castrated after the administration of lidocaine produced a significantly lower increase in plasma cortisol levels than piglets castrated after the administration of meloxicam. Similar observations were made by Hansson et al. [16], who found that the lidocaine groups produced significantly decreased scores in vocalization and defensive movements. A further study in 2005 showed that electroencephalographic, MAP and pulse rate responses to castration without analgesia were larger than the responses to castration after lidocaine injection [8]. The use of subcutaneous and intratesticular procaine in combination with flunixin provided significantly improved pain relief on the basis of plasma cortisol and vocalization parameters [17]. In contrast, other studies measured no differences in cortisol levels [18,19] when comparing piglet castration after administration of lidocaine and castration without analgesia.

The aim of this study was to investigate whether local anesthesia is able to achieve the elimination of nociception during surgical castration. Four different local anesthetics were evaluated on the basis of nociception-related physiological parameters and limb movements. Differentiating pain from emotions such as fear or anger is challenging in a conscious piglet. For this reason, a minimal anesthesia model using low doses of isoflurane was chosen. In this way, physiological reactions could be directly associated with nociception during castration.

## Materials and methods

The study was performed in compliance with the EU Directive 2010/63/EU for animal experiments and the German Animal Welfare Act (2018). All procedures were approved by the Ethical Committee for Animal Experiments of the Government of Upper Bavaria, Munich, Germany (Reference Number ROB-55.2-2532.Vet_02-19-11).

### Animals

Fifty-four hybrid German Landrace/German Large White x Pietrain male piglets aged 3 to 7 days (5.3±1.1 days) with weights above 1.4 kg (mean: 2.09 kg ± 0.4 kg) were included in this study. Pregnant sows were transferred to the animal husbandry unit of the Clinic for Swine (Oberschleißheim, Bavaria, Germany) at least two weeks before farrowing. Sows and piglets were housed according to the German Order for the Keeping of Productive Animals and the EU Directive 2010/63/EU for animal experiments. No teeth clipping, ear tagging, or tail docking was applied to the piglets. An oral bolus of iron (1 ml per piglet; URSOFERRAN^®^ 150 mg/ml, Serumwerk Bernburg AG, Bernburg, Germany) was given during the first ten hours of life. Piglets from 15 litters were randomly assigned to one of the six treatment groups (n = 9 per group).

### Study design

This was a randomized and double-blinded experimental study.

### Minimal anesthesia protocol

To measure nociception during injection and castration, a minimal anesthesia protocol was used to exclude disruptive factors such as fear and stress. The aim of the protocol was to keep the piglets in a hypnotic state with anesthesia (stage III plane I according to Guedel’s classification). Each group was induced with a dose of 5% isoflurane (Isoflurane Baxter vet., Baxter Deutschland GmbH, Unterschleißheim, Germany) in oxygen via a facial mask. Piglets breathed spontaneously, and inspiratory and expiratory isoflurane was assessed (Vamos^®^ plus, Dräger Medical Deutschland GmbH, Unterhaching, Germany).

**Fig 1:**
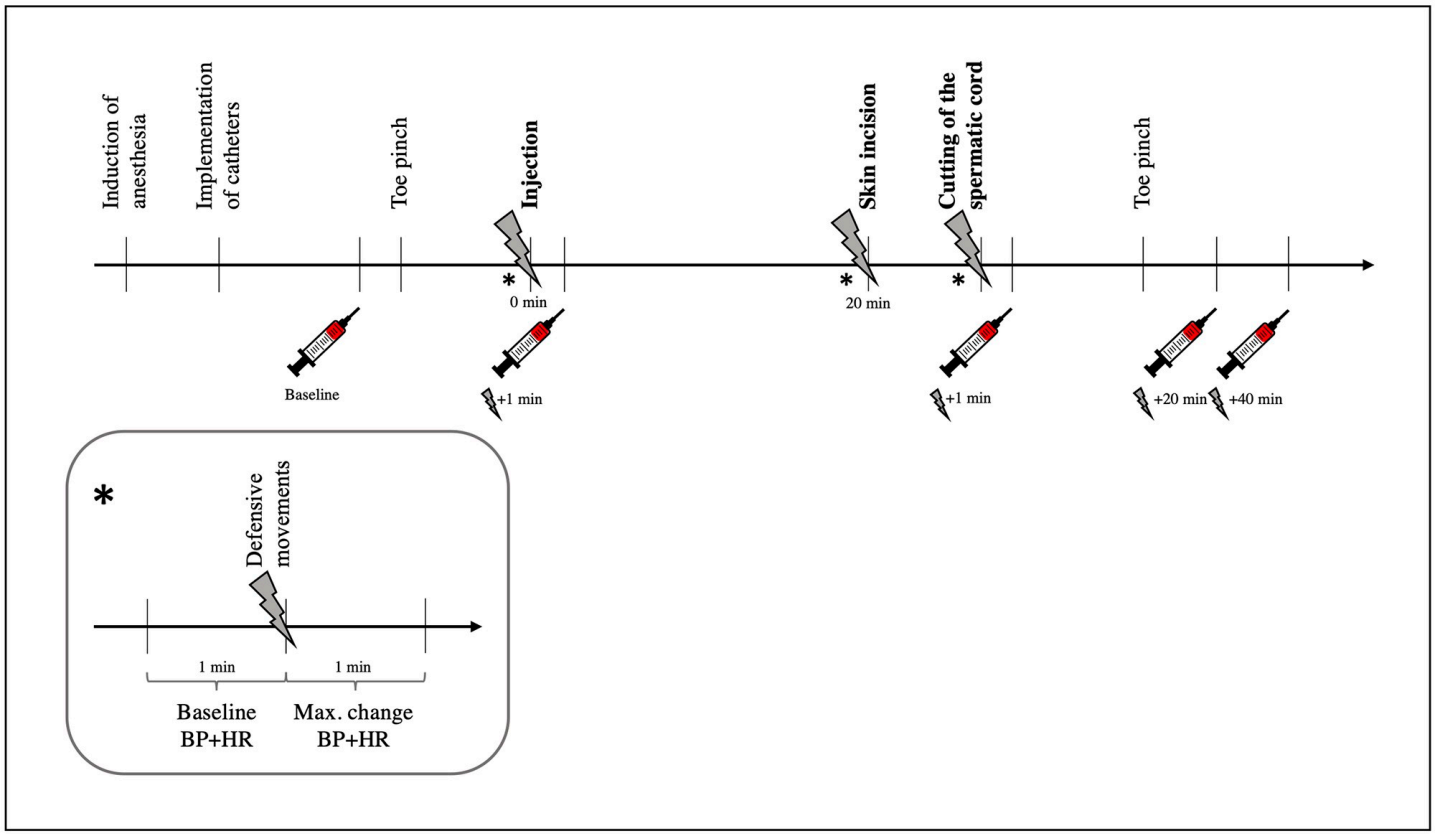
Overview of the experimental setup. The symbol 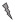 indicates the collection of blood samples. Baseline blood samples for all four blood parameters were taken 5 minutes after the implementation of the measurements. Blood samples for the analysis of epinephrine and norepinephrine were taken 1 minute after injection and 1 minute after the cutting of the spermatic cord. For cortisol and chromogranin A (CgA) measurements, blood samples were collected 20 and 40 minutes after the cutting of the spermatic cord. The symbol 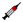 indicates a tissue damage event (injection, skin incision or cutting of the spermatic cord). * shows a more detailed overview of the time points of the measurements of BP, HR and limb movements during an event.

Following the necessary measurement preparations, adequate anesthetic depth was tested via the toe pinch reflex method, and end-tidal isoflurane was maintained at 1.69±0.3% with a flow of 3 L minute^-1^ oxygen. The reflex was triggered by closing a surgical clamp to a maximum of the first lock and for a maximum of 5 seconds in the interdigital space of the hind claw. To standardize the pressure as much as possible, the same pair of clamps was used by the same two persons throughout the study. A single reaction to the toe pinch, in the form of a slight withdrawal of the hind limb, was evaluated as appropriate anesthetic depth. If no reaction occurred at all, the isoflurane concentration was reduced by 0.2%, and after three minutes, another toe pinch was applied to the opposite hoof. Prolonged paddling and movement of the forelimbs, back or head were assessed as overshooting reactions, and isoflurane was increased by 0.2%. Subsequent to adjustment of the depth of narcosis, injection started after a three-minute waiting period. In the case of piglets moving due to fixation for injection or castration, isoflurane was increased by 0.2%, and two minutes passed before the intervention was started. Four minutes after castration, an additional toe pinch reflex was tested to verify the state of anesthesia. Anesthesia was maintained over 90 minutes to collect blood samples before the piglet was euthanized with an intravenous overdose of pentobarbital (Euthadorm 500 mg/ml Injektionslösung, CP Pharma, Burgdorf, Germany), and the spinal cord was removed for further study. An overview of the experimental setup is shown in Fig 1.

### Local anesthesia and castration

Animals were randomly assigned to the six treatment groups. The persons involved were double-blinded. All piglets, except the handling group (H), received a 0.5-ml intratesticular and 0.5-ml subscrotal injection 20 minutes prior to castration. Procaine has the longest onset time—20 minutes—of all tested local anesthetics; thus, this time was chosen to ensure comparability among all experimental groups. An automatic self-filling 1-ml syringe (HSW ECO-MATIC^®^, Henke-Sass, Wolf GmbH, Tuttlingen, Germany) with a 25G cannula (0.5x16 mm, B. Braun TravaCare GmbH, Hallbergmoos, Germany) was used for injection. The injections included procaine hydrochloride 4% (P, procaine) (procaine hydrochloride 4%—VMD, V.M.D. sa, Arendonk, Belgium), lidocaine hydrochloride 2% (L, lidocaine) (Xylocitin^®^ 2%, Mibe GmbH Arzneimittel, Brehna, Germany), bupivacaine hydrochloride 0.5% (B, bupivacaine) (Bupivacain 0.5% JENAPHARM, Mibe GmbH Arzneimittel, Brehna, Germany) and mepivacaine hydrochloride (M, mepivacaine) (Mepidor^®^ 20 mg/ml solution for injection for horses, Richter Pharma AG, Wels, Austria). As a positive control, one group received injections with only sodium chloride 0.9% before castration (NaCl, castration without pain relief). As a negative control, another group underwent simulated injection and castration (H, handling). Surgical castration (groups P, L, B, M, NaCl) was performed using two vertical scrotal incisions. To evaluate the noxious stimulus of skin incision and the noxious stimulus of cutting the spermatic cord separately, a period of 2 minutes for stabilization of hemodynamic parameters was provided before the spermatic cord of the right and left testicle were severed using an emasculator. Piglets in group H were only prepared for castration without the occurrence of any painful procedure. Isoflurane anesthesia and preparations and measurements (blood parameters, BP, HR, movements, etc.) were carried out as in the other experimental groups. The only difference in group H was that injection and castration were only simulated. More precisely, handling of the piglets was performed by one person for injection and castration. Instead of performing an injection, the testis was just fixed and gently touched with a needle cap. Castration was simulated by fixing the testis and gently touching it with the blunt back of the scalpel handle (castration).

### Measurements

#### Implementation of physiologic measurements

In this study, it was assumed that all piglets experienced similar stress from the induction procedure and the establishment of the measurements. Twenty minutes before anesthetic induction, a local anesthetic cream (Emla^®^, AstraZenecea GmbH, Wedel, Germany) was applied to the piglet’s ventral neck to numb the skin for the establishment of venous and arterial access. After anesthetic induction, piglets were placed in a supine position on the heated ground. Eye ointment (Bepanthen Augen- und Nasensalbe, Bayer Vital GmbH, Leverkusen, Germany) was administered, and cotton wool was placed in the external auditory canal to minimize physiological reactions to background noises. Oxygen saturation and HR were measured via a pulse oximeter (2500A VET, Nonin Medical Inc., Plymouth, MN, USA) placed at the base of the piglet’s tail. Body temperature was recorded with the help of a rectal temperature probe and remained constant by providing an additional heat supply. Respiratory frequency and end-tidal carbon dioxide were monitored by capnometry (Vamos^®^plus, Dräger Medical Deutschland GmbH, Unterhaching, Germany) via a breathing filter connected to the mask. Skin at the arterial and venous access site was injected with a maximum of 0.3 ml lidocaine (lidocaine 2%, bela-pharm Arzneimittelfabrik, Vechta, Germany). The left carotid artery and jugular vein were visualized with gentle preparation, and a catheter (3F, Balt extrusion, Montmorency, France) was introduced to both vessels. For invasive BP measurement, a micro tip catheter (SPR-407 Mikro-Tip^®^, Millar, Inc., Houston, TX, USA) was placed in the carotid artery. Before each measurement, the catheter was calibrated. Therefore, the tip of the catheter was placed just beneath the surface of a water bath that was heated to the piglet’s body temperature (39 °C). Systolic, diastolic and MAP and an electrocardiogram (ECG) were recorded continuously with appropriate hardware modules and corresponding software (PLUGSYS module, Transducer Amplifier module TAM, heart rate module, HAEMODYN software, Hugo Sachs Elektronik–Harvard Apparatus GmbH, March-Hugstetten, Germany). Baseline values were collected over one minute prior to the “injection” and “castration” events. Mean values and standard deviations of BP and HR were calculated. Subsequently, within one minute after every event, the maximum change from baseline was selected, and the percent deviation from baseline was determined.

#### Blood samples

To maintain hydration, a crystalloid intravenous infusion (Sterofundin Iso, B. Braun Melsungen AG, Melsungen, Germany) was administered at a rate of 4 ml/kg/h, and the extracted blood volume was directly substituted. Blood samples (max. 1.8 ml each sample) were taken via the venous port at the following time points. Baseline values for all parameters were taken five minutes after placement of the catheters and prior to the first toe pinch reflex test. For the analysis of catecholamines, EGTA-plasma tubes were prechilled on ice. Samples were collected one minute after injection and one minute after cutting the spermatic cord. Serum samples for cortisol and CgA were taken 20 and 40 minutes after castration (cutting of the spermatic cord). Blood samples for catecholamines were centrifuged (5 minutes, 3000 U/min, 4 °C) immediately after collection. Serum samples were stored for one hour at room temperature before centrifugation (10 minutes, 3000 U/min, 4 °C). Collected plasma samples were stored at -80 °C, and serum samples were stored at -20 °C until analysis by a certified laboratory.

#### Limb movements

During injection, incision and spermatic cord transection, each piglet was immobilized by the same person, who held the piglet in a supine position with both hands under the back and the hindquarters. This ensured that both the movements of the four limbs and tension of the back could be perceived and assessed. In addition, each event was filmed and evaluated. To assess the quality of limb movements for every piglet and event, a specific score was established based on Berchtold et al. [20] and Hug et al. [21]. Both front and hind limbs as well as the spine/back were assessed individually for each testicle. A maximum score of 28 points (14 points per testicle) could be recorded per event (injection, skin incision, cutting of the spermatic cord) (Table 1).

**Table 1:** Scoring system adapted from Berchtold [20] and Hug et al. [21] to assess pain during castration on the basis of limb movements.

**Limb score (each limb is scored separately)**	**Number of movements**
0	No reaction
1	One movement
2	Two to three movements
3	More than three movements, long-lasting movements
Highest limb score per testicle	12
**Back/spine score**	**Number of movements**
0	No reaction
1	Muscle contraction
2	Movements
Highest back/spine score per testicle	2
Maximum score per testicle:	14
Maximum score per event:	28

#### Statistical analysis

For the statistical analysis, groups were tested for changes in BP and HR, age and weight at castration. The distribution of all continuous parameters was tested using the Shapiro-Wilk normality test. For all normally distributed parameters, a parametric one-way ANOVA test was performed. Pairwise t-test comparisons between groups with post-hoc Bonferroni corrections for multiple testing followed by ANOVA. For nonnormally distributed parameters, a Kruskal-Wallis test was performed. Pairwise Wilcoxon tests for comparisons between parameters with post-hoc Bonferroni corrections for multiple testing were followed by the Kruskal-Wallis test. Blood parameters were analyzed using a mixed-effects model due to the presence of repeated measures. Blood parameter data were log-transformed prior to analysis due to nonnormally distributed and heteroscedastic residuals. Pairwise comparisons of factor combinations in the mixed-effects model were conducted via estimated marginal means with post hoc Bonferroni corrections. Statistical significance was considered at p ≤ 0.05. All statistical analyses were performed using R version 3.6.1 (2019-07-05) [22].

## Results

The mean ± standard error of the mean (SEM) body weights of the piglets in each treatment group were as follows: H: 2.2 ± 0.5; NaCl: 2.1 ± 0.6; P: 1.8 ± 0.3; L: 2.5 ± 0.5; B: 2.1 ± 0.4; and M: 2.2 ± 0.4 kg. The mean ± SEM ages of the piglets in each treatment group were as follows: H: 5.3 ± 1.2; NaCl: 4.9 ± 1.3; P: 5.1 ± 1.2; L: 5.3 ± 1.4; B: 5.6 ± 1.0; and M: 5.5 ± 1.0 days. Mean body weight and age did not significantly differ between the treatment groups.

### Blood pressure

**Fig 2:**
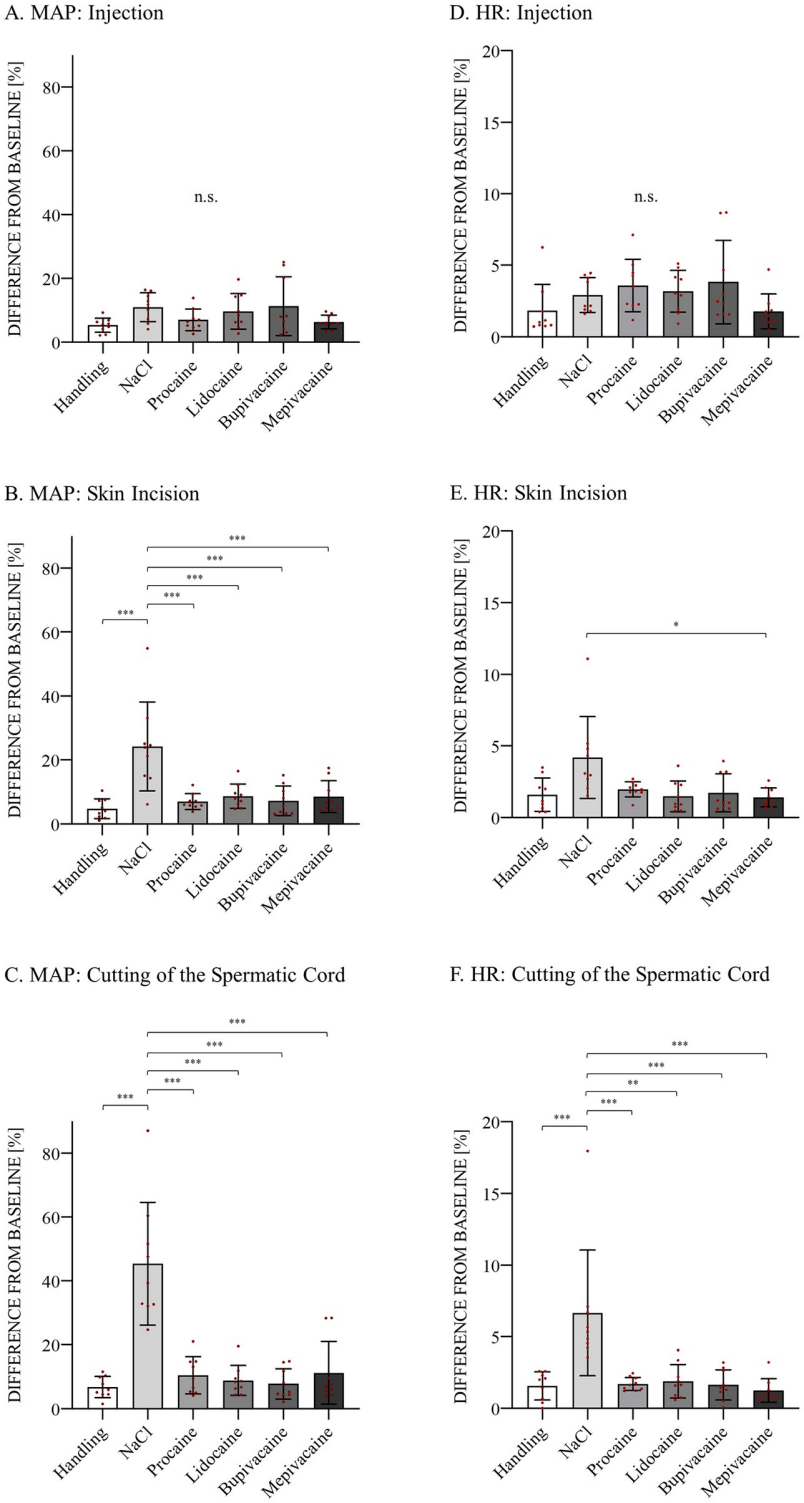
Percent change in mean arterial blood pressure (MAP) and Heart Rate (HR). Twenty minutes prior to castration, a 0.5-ml i.t. injection and a 0.5-ml subscrotal injection were administered in each testis. The six bars illustrate the maximum percent change from baseline within 1 minute after injection (A, D), skin incision (B, E) and cutting of the spermatic cord (C, F) in the different experimental groups. Handling = no injection and no castration, only simulation of the procedures; NaCl = injection of sodium chloride (NaCl) and castration; Procaine = injection of procaine 4% and castration; Lidocaine = injection of lidocaine 2% and castration; Bupivacaine = injection of bupivacaine 0.5% and castration; Mepivacaine = injection of mepivacaine 2% and castration. Values shown are means ± SEMs. Statistical significance is indicated by * p ≤ 0.05, ** p < 0.01, *** p < 0.001; n.s. = no significance among groups.

The mean MAP of all piglets was 55.68 ± 7.15 mmHg. Fig 2 shows the mean of the maximum changes in MAP after injection, skin incision and cutting of the spermatic cord in each treatment group. During injection, no significant difference among the groups was detected. Local anesthetic groups P (p < 0.001), L (p < 0.001), B (p < 0.001) and M (p < 0.001) showed statistically significant reductions in maximum MAP changes compared to the NaCl group after skin incision. The deviation from baseline in the NaCl group was 24%. This was the highest value observed during skin incision and was also significantly different from that in the H group (p < 0.001). Regarding cutting of the spermatic cord in local anesthetic groups P (p < 0.001), L (p < 0.001), B (p < 0.001) and M (p < 0.001), the deviation in the maximum MAP change was significantly decreased compared to that in the NaCl group. Between the local anesthetic groups and compared to the H group, no significant difference was detected. Similar to skin incision, NaCl had the highest value (45%) after cutting the spermatic cord and was significantly increased compared to H (p < 0.001).

### Heart rate

No significant differences in HR deviation were detected among the treatment and control groups after injection. The HR after skin incision in group M was significantly decreased compared to that in group NaCl (p < 0.001). There was no significant difference in the mean HR among the other groups. In general, a highly significant change in HR from baseline after cutting of the spermatic cord was discovered in groups P (p < 0.001), L (p = 0.002), B (p < 0.001) and M (p < 0.001) compared to that in group NaCl. Additionally, NaCl was associated with a significant increase compared to H (p < 0.001) (Fig 2).

### Blood samples

#### Cortisol

Plasma cortisol concentrations in piglets at time points before surgical castration and 20 and 40 minutes after castration are shown in Table 2. Comparison among the experimental groups revealed no significant differences in plasma cortisol concentrations at any time point. Within the groups, 20 (H: p < 0.001, NaCl: p < 0.001, P: p < 0.001, L: p < 0.001, B: p < 0.001, M: p < 0.001) and 40 (H: p < 0.001, NaCl: p < 0.001, P: p < 0.001, L: p < 0.001, B: p < 0.001, M: p < 0.001) minutes after castration, increased cortisol levels compared to baseline levels were measured.

**Table 2:** Influence of treatment on serum cortisol concentrations 20 and 40 minutes after the castration of piglets.

	Handling	NaCl	Procaine	Lidocaine	Bupivacaine	Mepivacaine
**Cortisol (μg/L)**						
Baseline	65 ± 19.24	71.44 ± 35.73	67.22 ± 14.25	72.56 ± 28.06	67.56 ± 21.56	68.00 ± 13.48
20 m p. castr.	104.78 ± 25.40 ***	92.89 ± 32.79 ***	98.11 ± 31.03 ***	101.33 ± 34.67 ***	107.33 ± 27.73 ***	97.89 ± 19.90 ***
40 m p. castr.	108.89 ± 24.06 ***	91.78 ± 40.90 ***	89.00 ± 33.82 ***	105.44 ± 35.96 ***	110 ± 33.31 ***	98.78 ± 27.03 ***

Mean ± SEM serum cortisol concentrations (μg/L) are shown for the different treatment groups. Values shown are means ± SEMs. A statistically significant change from baseline is indicated by * p ≤ 0. 05, ** p < 0.01, *** p < 0.001, n.s. = no significance.

#### Catecholamines

Epinephrine and norepinephrine concentrations in piglets measured before castration as well as one minute after injection and castration are shown in Table 3. No significant differences were found among the groups at any time point. Baseline measurements for both epinephrine and norepinephrine greatly differed among the experimental groups. A significant increase in epinephrine from baseline to castration (H: p < 0.001, NaCl: p < 0.001, P: p < 0.001, L: p < 0.001, B: p < 0.001, M: p < 0.001) and injection to castration (H: p < 0.001, NaCl: p < 0.001, P: p < 0.001, L: p < 0.001, B: p < 0.001, M: p < 0.001) was measured within all groups. No significant differences were found in norepinephrine measurements.

**Table 3:** Influence of local anesthesia on plasma catecholamines after the injection and castration of piglets.

	Handling	NaCl	Procaine	Lidocaine	Bupivacaine	Mepivacaine
**Epinephrine (ng/L)**						
Baseline	199.67 ± 142.83	126.56 ± 75.43	114.11 ± 67.65	174.33 ± 161.30	153.67 ± 88.31	107.67 ± 62.02
Injection	230.44 ± 197.80 n.s.	113.89 ± 69.39 n.s.	103.89 ± 48.65 n.s.	129.44 ± 103.60 n.s.	165.13 ± 86.84 n.s.	115.00 ± 67.25 n.s.
Castration	262.11 ± 237.10 *** ◆◆◆	142.11 ± 105.42 *** ◆◆◆	245.33 ± 155.45 *** ◆◆◆	237.44 ± 240.70 *** ◆◆◆	185.56 ± 106.41 *** ◆◆◆	207.33 ± 175.51 *** ◆◆◆
**Norepinephrine (ng/L)**						
Baseline	359 ± 250.74	244.33 ± 152.34	435.67 ± 263.52	424.22 ± 423.93	284.78 ± 157.33	204.11 ± 164.79
Injection	436.89 ± 405.26 n.s.	273.78 ± 152.32 n.s.	383.44 ± 205.71 n.s.	341.67 ± 361.57 n.s.	430,625 ± 382.24 n.s.	242.00 ± 279.25 n.s.
Castration	341.56 ± 246.52 n.s.	263.67 ± 88.61 n.s.	620.89 ± 422.36 n.s.	349.11 ± 405.10 n.s.	420.67 ± 406.96 n.s.	231.78 ± 217.45 n.s.

Mean ± SEM plasma epinephrine and norepinephrine concentrations (μg/L) are shown for the different treatment groups. Baseline blood samples were taken 5 minutes after placement of the catheters. The remaining samples were taken 1 minute after injection and cutting of the spermatic cord in each case. Values shown are means ± SEMs. A statistically significant change from baseline is indicated by * p ≤ 0. 05, ** p < 0.01, *** p < 0.001, n.s. = no significance. A statistically significant change from injection is indicated by ◆ p ≤ 0. 05, ◆◆ p < 0.01, ◆◆◆ p < 0.001, n.s. = no significance.

#### Chromogranin A

Plasma CgA concentrations in piglets at time points before surgical castration and 20 and 40 minutes after surgery are shown in Table 4. None of the measurements among the groups at any time point were significant.

**Table 4:** Influence of treatment on serum CgA 20 and 40 minutes after the castration of piglets.

	Handling	NaCl	Procaine	Lidocaine	Bupivacaine	Mepivacaine
**Chromogranin A (μg/mL)**						
Baseline	0.90 ± 0.39	0.68 ± 0.25	0.97 ± 0.61	0.73 ± 0.25	0.96 ± 0.63	0.88 ± 0.53
20 m p. castr.	1.28 ± 1.44 n.s.	0.66 ± 0.26 n.s.	0.65 ± 0.45 n.s.	0.72 ± 0.26 n.s.	0.73 ± 0.46 n.s.	0.94 ± 0,70 n.s.
40 m p. castr.	0.85 ± 0.28 n.s.	0.63 ± 0.11 n.s.	0.57 ± 0.29 n.s.	0.80 ± 0.33 n.s.	0.78 ± 0.50 n.s.	0.87 ± 0.53 n.s.

Mean ± SEM serum CgA concentrations (μg/L) are shown for the different treatment groups. Baseline blood samples were taken 5 minutes after placement of the catheters. Values shown are means ± SEMs. A statistically significant change from baseline is indicated by * p ≤ 0. 05, ** p < 0.01, *** p < 0.001, n.s. = no significance.

#### Limb movements

**Fig 3:**
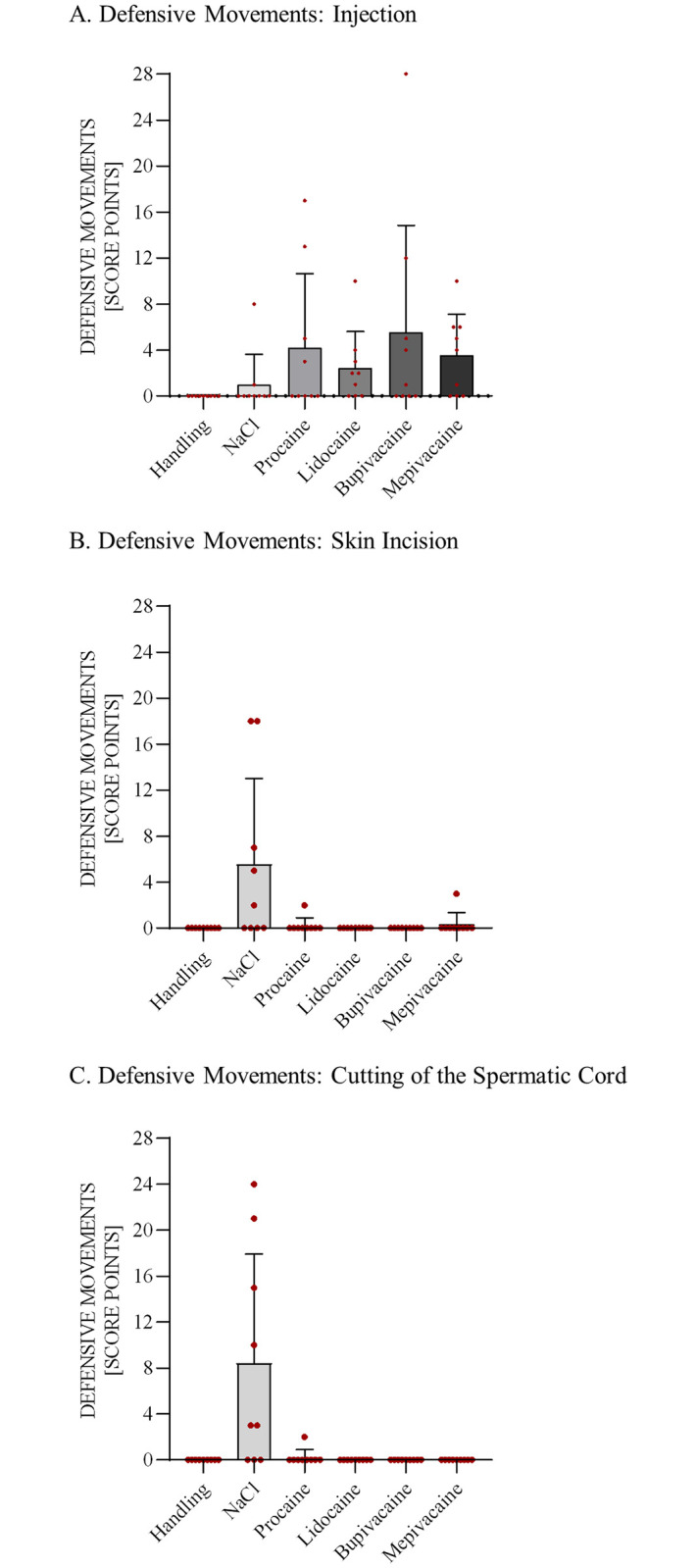
Limb movement scores during injection, skin incision and cutting of the spermatic cord. Limb movements were observed during injection (A), skin incision (B) and cutting of the spermatic cord (C). The score was applied to analyze the intensity of limb movements: handling = neither injection nor castration, only simulation of the procedures; NaCl = castration without preoperative analgesia; procaine, lidocaine, bupivacaine, and mepivacaine = castration under preoperative local anesthetic.

During injection, B scored highest (5.6), followed by P (4.2), M (3.4), L (2.4) and NaCl (1). During skin incision, the NaCl group had the highest score, with 5.6 points on average, followed by the M (0.3) and P (4.2) groups. In the L and B groups, no limb movements were registered. After the spermatic cord was cut, the NaCl group reached a score of 8.4, and the P group had a score of 0.2. The L, B and M groups did not move at all (Fig 3).

During injection, two piglets in the NaCl group moved, while no piglets in the H group showed signs of limb movements. The piglets injected with a local anesthetic moved more often (P: n = 4; L: n = 6; B: n = 5; M: n = 6) than those in the control groups. Skin incision prompted movements only in piglets in the NaCl (n = 5), P (n = 1) and M (n = 1) groups. Similarly, during the cutting of the spermatic cord, the highest number of piglets showing limb movements was observed in the NaCl group (n = 6). Aside from one piglet in the P group, no limb movements were detected in piglets in the other experimental groups.

## Discussion

The present study reconfirmed that surgical castration is a highly painful method [23,24]. A minimal isoflurane anesthesia model was used to assess acute physiological alterations to validate the analgesic effects of four local anesthetics during castration. As nociception-related parameters, invasive BP and HR as well as blood concentrations of cortisol, epinephrine, norepinephrine and chromogranin A were measured. In addition, limb movements of the piglets were studied. As pain-related physiological responses are easily influenced by stress and fear, it is difficult to discriminate pain from handling stress [7]. Therefore, to measure pure nociception, light isoflurane anesthesia was administered. Isoflurane is a volatile agent with no analgesic effects at low dosages. Even if it has moderate cardiodepressive effects, in the clinical range, isoflurane shows no influence on the magnitude of BP or HR changes in response to noxious stimuli [25,26]. Therefore, the use of isoflurane has a low impact on studying nociception.

Our results showed that local anesthesia significantly reduced BP and HR changes after skin incision and cutting of the spermatic cord compared to castration without any pain medication. MAP was the most significant parameter associated with noxious stimuli during castration. This finding is in line with a study that observed that MAP was the most sensitive nociceptive indicator for pigs under isoflurane anesthesia [10]. Haga and Ranheim [8] found similar results when measuring invasive arterial BP during castration with lidocaine performed under halothane anesthesia. During all three steps of the castration procedure, H induced minimal BP changes that were in the range of normal BP fluctuations. Most notably, BP changes in the four local anesthetic groups were comparable to the change in the H group. This suggests that local anesthesia reduces nociception induced by surgical castration to a level of unpleasantness similar to that felt by piglets handled in the inguinal region under isoflurane anesthesia. Castration without any pain relief provoked the highest increase in BP. Bova et al. [27] determined that if a BP increase of more than 20% occurs during surgical intervention, relevant pain must be assumed, and rescue analgesia may be necessary. In the NaCl group, BP increased by 24% during skin incision, and an average increase of 45% was observed while cutting the spermatic cord. Both events have to be considered highly painful, and it was reconfirmed that manipulating and cutting the spermatic cord is the most painful part of castration [28].

In approximately half of the animals, as the maximum deviation from baseline, a decrease in BP and HR was observed in response to manipulation during the castration procedure. A decrease in BP did not necessarily represent a decrease in HR in the same piglet. Similar observations were made in piglets, lambs and calves under halothane anesthesia undergoing zootechnical procedures [8,25,29]. Van Lieshout et al. [30] attributed this paradoxical effect to a vasovagal response to noxious stimuli. The occurrence of bradycardia and hypotension during severe pain caused by the stretching of abdominal or perineal viscera has been described in the literature [31]. A similar type of pain could be induced through the stretching of the spermatic cord, and therefore the peritoneum, in the castration process. Above a certain concentration, halothane induces a depressor hemodynamic response to noxious stimuli in rats; however, this effect has not been described in animals under isoflurane anesthesia [32]. Other explanations could be alterations in respiratory function or increases in peripheral vascular resistance [29,33]. As the decreases in BP and HR were directly related to a stimulus, we believe that it is reasonable to value both a large increase and a large decrease as a sign of nociception.

All four local anesthetics provided a reduction in limb movements during skin incision and cutting of the spermatic cord. The NaCl group showed the most limb movements during castration, and piglets in the H group did not move at any time point. Several other studies observed a beneficial effect regarding limb movements in piglets castrated under local anesthesia [2,9,16]. It must be noted that all these studies were carried out in conscious piglets. A point of criticism could be that limb movements are hard to evaluate under isoflurane anesthesia. Volatile anesthetics at certain concentrations are known to block movements in response to noxious stimuli [34]. To ensure that a reaction to noxious stimuli was still possible, the isoflurane concentration was individually titrated via reaction to a toe pinch. Toe pinch was chosen because Eger et al. [35] found that toe clamping is a painful stimulus and prompts the best reflex to determine anesthetic depth in piglets [36]. As the toe pinch was carried out by a human, the pressure applied might be inconsistent. To minimize this variance in applied pressure and localization, the reflex was always tested by the same two persons, and the same clamp was used. Only when a reaction to a toe pinch was observed was the experiment continued. It cannot be fully excluded that in some animals, anesthesia might have blocked some reactions; in the NaCl group, only six out of nine piglets exhibited limb movements. Nevertheless, the limb movements are in line with the BP and HR results, which suggests this model produced reliable results.

Regarding the injection technique used in this study, it must be noted that piglets in all experimental groups receiving i.t. and subscrotal injections showed clear limb movement responses. Additionally, two animals in the NaCl group reacted to the injection. BP and HR in injected piglets were higher than those in the handling group, but no significant difference was found. These reactions in response to injection indicate that injections into the testis are painful. An explanation regarding why piglets injected with a local anesthetic reacted more could be that local anesthetics are acidic solutions that could provoke a burning sensation in the injected area. This suggests that in addition to the injection itself, the pH of the applied solution plays a role as a pain trigger. Hanna et al. [37] showed that pain induced by the injection of local anesthetics is caused by multiple factors and can be reduced by adding bicarbonate. This must be further discussed if intratesticular injection is to meet the requirements of the German welfare act, which requests the total elimination of pain during the surgical procedure. Perhaps using lower volume, buffered local anesthetics or different injection techniques could further improve injection pain. Hogan et al. [38] suggests warming the local anesthetic to reduce injection pain. The optimization of the injection technique and the verification of whether the same analgesic effects can be achieved by these methods are part of further studies.

Regarding the analysis of stress hormones after castration after the administration of local anesthesia, the results of previous studies are inconclusive. Sutherland et al. [39] did not find a reduced cortisol response after castration with the administration of local anesthetics compared to piglets castrated without anesthesia. In contrast, in two other studies, the serum cortisol levels of piglets treated with local anesthetics were even higher than those in the control groups [40,41]. Rauh et al. [9] even observed an increase in epinephrine and norepinephrine in piglets injected with 2% procaine, and Hofmann et al. [42] observed similar effects of procaine on cortisol concentrations and 1% lidocaine on chromogranin A levels. However, due to the use of different local anesthetics, concentrations, application times and types of application in the studies, a direct comparison is not possible.

In the present study, these findings could not be confirmed. Injection into the testis and castration did not cause significant changes in the stress hormone concentrations of cortisol, CgA, epinephrine or norepinephrine in the experimental groups. In the handling group, even higher increases than those in the NaCl group were recorded.

An explanation for the failure of blood hormones to illustrate the nociception experienced by the piglets in our model may be that the procedures were performed under isoflurane anesthesia. The baseline blood samples were taken 31 minutes after the induction of anesthesia (31.04 ± 7.1 minutes). We assume that the high baseline cortisol level, compared to other studies [41,43,44], could be caused by basic stress due to factors such as handling or induction of anesthesia through a mask.

However, the low catecholamine baseline could be explained by the absence of acute stress caused by handling. The baseline was comparable to studies in which blood samples were taken without stress via a venous port from piglets without anesthesia [45] or without a venous port but under isoflurane anesthesia [44].

Furthermore, in humans, increases in catecholamine and cortisol concentrations under isoflurane anesthesia have been described, and these reactions could also appear in piglets [46]. However, significant increases in cortisol concentrations within groups were observed, suggesting a general, group-independent increase in stress under this model.

Whereas different concentrations and application techniques of lidocaine [2,3,8,9,15–19,21,39,41,47–56] and procaine [9,17,41,42,57–59] have been excessively studied in the context of piglet castration, bupivacaine was analyzed only in combination with lidocaine by Bonastre et al. [60]. To the best of our knowledge, mepivacaine, an approved drug in several European countries for intraarticular and epidural injections in horses, has never been used in studies examining piglet castration. Although all of the local anesthetics used in our study were administered in high doses, no cardiovascular or pulmonary side effects were observed. To speed up the onset of action and to prolong the duration of action, epinephrine is often added to local anesthetics. As a pilot study showed that i.t. in combination with subscrotally injected epinephrine distorted the measurements of the hemodynamic parameters (Fig I in S1 Appendix) and plasma epinephrine (Fig II in S1 Appendix), no epinephrine was added to the analyzed substances. No significant differences were found between the four local anesthetics, indicating that all of them obtained sufficient analgesic effects under light isoflurane anesthesia, reducing acute nociceptive castration pain.

A limitation of the present study is that nociception is only assessed on the basis of acute cardiovascular and humoral responses and limb movements. To obtain a more complete picture of nociception, neural nociceptive processing must be included in the evaluation by studying Fos protein expression in the spinal cord and EEG responses. These data were also collected and will be published separately due to their high complexity (manuscript in preparation).

Further comparisons between the tested substances are planned in awake piglets considering behavior and postcastration pain, where characteristics such as duration of action and time of onset play a more important role. All these results must be taken into account before a final evaluation of local anesthesia as a method to eliminate pain during piglet castration is possible.

## Conclusions

Our study demonstrates that the nociception induced by intratesticular and subcutaneous injection and castration can be reliably quantified by measuring cardiovascular responses and limb movements under isoflurane anesthesia. An intratesticular injection with a subcutaneous depot of a local anesthetic prior to castration reduces nociception-related cardiovascular responses and limb movements compared to castration without a local anesthetic.

Our findings confirm the analgesic effect of the investigated local anesthetics caused by blocking nociception during castration in our experimental setup in piglets. However, the injection of local anesthetics caused visible nociception, as shown by increased limb movements.

It must be mentioned that our study investigated the analgesic effects of local anesthesia during castration in piglets under light narcosis, which means that nociception was evaluated with the exclusion of stress and fear. If conscious piglets are castrated, then these factors will influence the piglets’ behavior and perception of pain. Whether this method is also practicable and effective in conscious piglets and how the different local anesthetics influence wound healing and post-castration behavior will be elucidated in further studies.

## Supporting information

S1 Appendix Systemic influence of intratesticularly and subscrotally injected epinephrine. (DOCX)
