# Supplementary material for: Local anesthesia in piglets undergoing castration—A comparative study to investigate the analgesic effects of four local anesthetics on the basis of acute physiological responses and limb movements
Source: PLoS One. 2020 Jul 30;15(7):e0236742. doi: 10.1371/journal.pone.0236742 (PMC7392247; doi:10.1371/journal.pone.0236742)
Supplement: S1 Appendix — (DOCX) [file pone.0236742.s001.docx]

### S1 Appendix. Systemic Influence of Intratesticularly and Subscrotally Injected Epinephrine.

To analyze the influence of epinephrine on BP, HR and plasma epinephrine concentrations, a pilot study was carried out. The experimental setup corresponded to the main study design. The 0.5 ml i.t. and 0.5 ml subscrotal injections contained either NaCl (NaCl; n=6) or NaCl mixed with epinephrine (0.02 mg/ml) (NaCl+Epi; n=6). After 20 minutes, castration was performed.

Mean of the maximum changes in MAP and HR after injection, skin incision and cutting of the spermatic cord in each treatment group were calculated. For statistical analysis the distribution was tested with Shapiro-Wilk normality test. For normally distributed data a Welch Two Sample t-test and for nonnormally distributed data a Wilcoxon rank-sum test was used. During injection, no significant difference among the groups was detected. NaCl+Epi group showed significantly increased MAP after skin incision (p = 0.026). After cutting of the spermatic cord an increase in NaCl+Epi (p = 0.066) was observed as well as a significant increase in HR (p = 0.011) (Fig I S1).


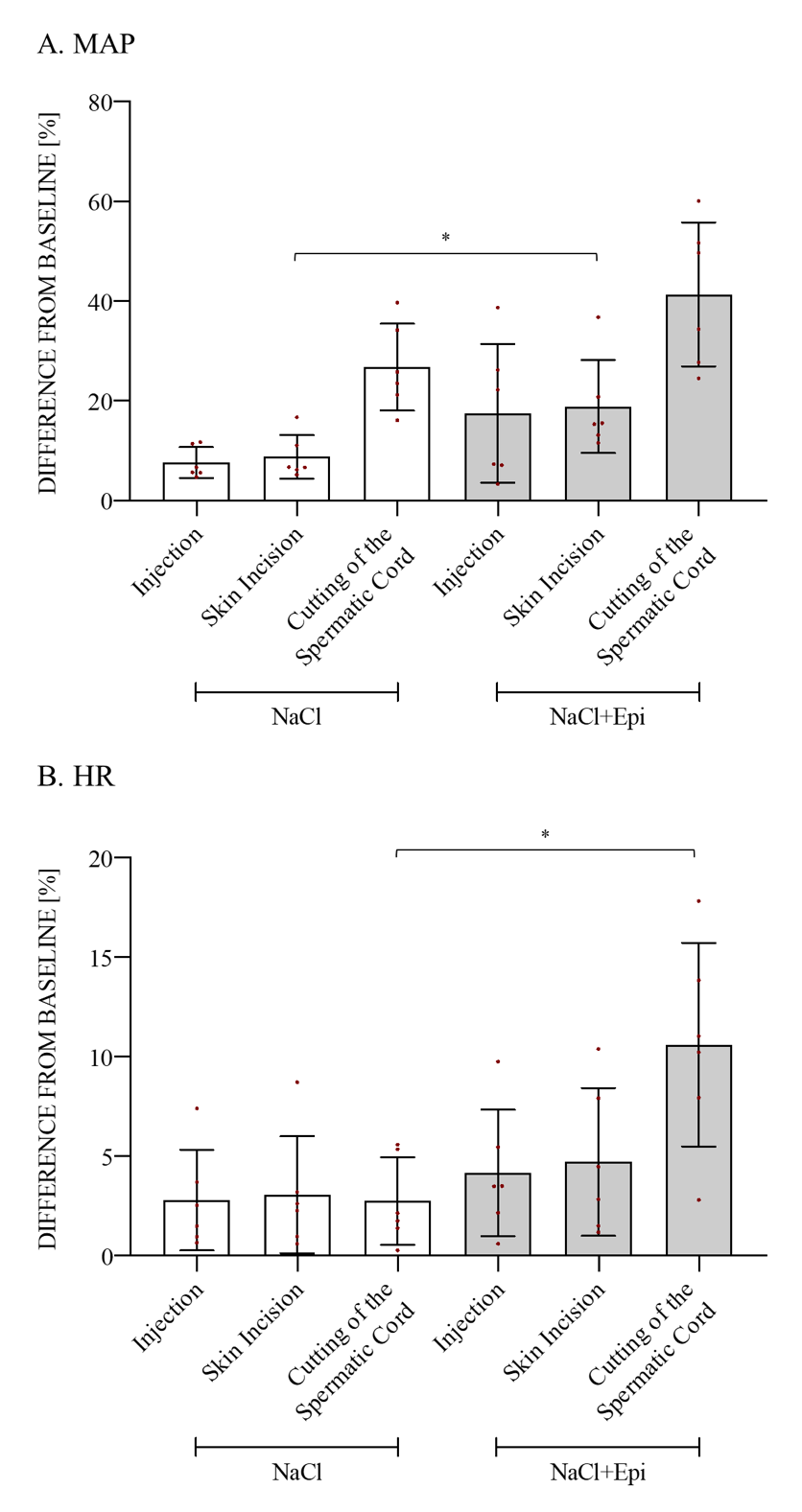


**Fig I S1. Percent Change in Mean Arterial Blood Pressure (MAP, A) and Heart Rate (HR, B) after Intratesticular Epinephrine Injection.** Twenty minutes prior to castration, an intratesticular injection of 0.5 ml and an additional 0.5 ml subcutaneous depot were administered to each testis. NaCl = injection of sodium chloride 0.9 %, NaCl+Epi = injection of sodium chloride 0.9 % with 0.02 mg/ml epinephrine. n= 6 per group. Values shown are means ± SEMs. Statistical significance is indicated by * p ≤ 0.05, ** p < 0.01, *** p < 0.001; n.s. = no significance.

Fig II S1 shows increases in plasma epinephrine concentrations in piglets one minute after i.t. and subscrotal injection with epinephrine and castration compared to the NaCl controls. For statistical anlaysis data were log-transformed and a mixed-effects model was used. Post-hoc pairwise comparisons were adjusted using Bonferroni correction of p-values. NaCl (p = 0.015) and NaCl+Epi (p = 0.015) showed a significant increase after cutting of the spermatic cord but no significant difference between groups was observed.

Based on the results of the pilot study, it was decided to use local anesthetics without epinephrine in the main study. In this way, it could be ensured that the measured parameters were not falsified by added epinephrine.

**
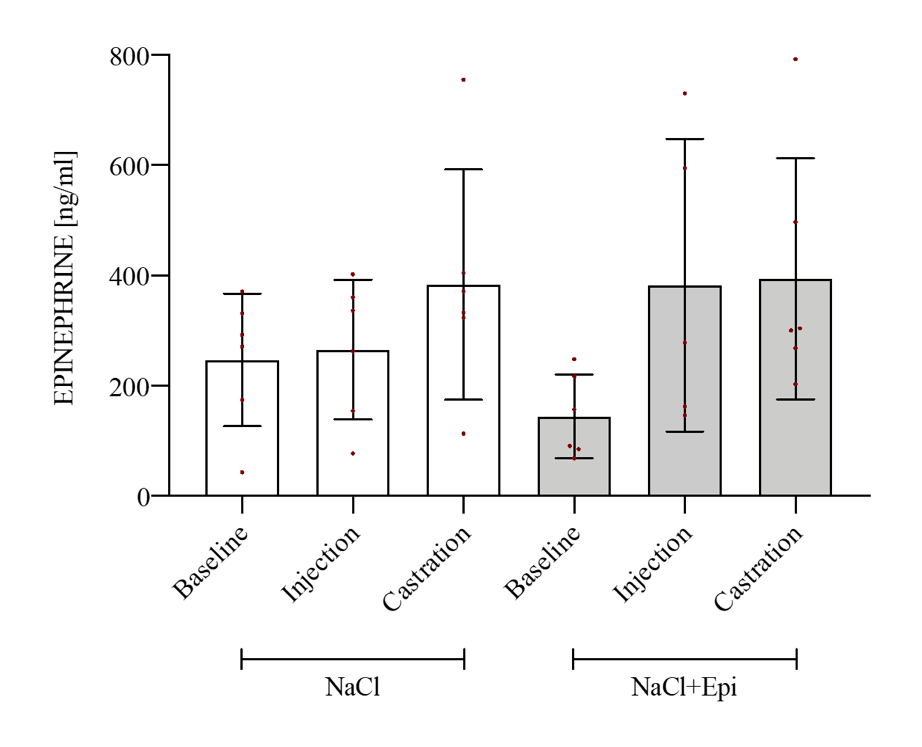
**

**Fig II S1. Percent Change in Plasma Epinephrine Concentrations One Minute after Intratesticular Epinephrine Injection and Castration.** NaCl = injection of sodium chloride 0.9 %, NaCl+Epi = injection of sodium chloride 0.9 % with 0.02 mg/ml epinephrine. n= 6 per group. Values shown are means ± SEMs.
